# Survival after partial heart transplantation in a piglet model

**DOI:** 10.1038/s41598-024-63072-1

**Published:** 2024-05-29

**Authors:** Cathlyn K. Medina, Mary E. Moya-Mendez, Berk Aykut, Sydney Jeffs, Lillian Kang, Amy Evans, Lauren E. Parker, Stephen G. Miller, Kristi L. Helke, Douglas M. Overbey, Joseph W. Turek, Taufiek Konrad Rajab

**Affiliations:** 1 Department of Surgery, Duke University Medical Center, Durham, NC USA; 2 Division of Cardiovascular Perfusion, Department of Clinical Sciences, Duke University, Durham, USA; 3 Department of Pediatrics, Duke University Medical Center, Durham, NC USA; 4 Department of Comparative Medicine, Medical University of South Carolina, Charleston, SC USA; 5 Department of Surgery, Arkansas Children’s Hospital, 1 Children’s Way, Little Rock, AR 72202 USA

**Keywords:** Congenital heart disease, Heart transplant, Pulmonary valve, Congenital cardiac surgery, Translational research, Congenital heart defects

## Abstract

Partial heart transplantation (PHT) is a novel surgical approach that involves transplantation of only the part of the heart containing a valve. The rationale for this approach is to deliver growing heart valve implants that reduce the need for future re-operations in children. However, prior to clinical application of this approach, it was important to assess it in a preclinical model. To investigate PHT short-term outcomes and safety, we performed PHT in a piglet model. Yorkshire piglets (n = 14) were used for PHT of the pulmonary valve. Donor and recipient pairs were matched based on blood types. The piglets underwent PHT at an average age of 44 days (range 34–53). Post-operatively, the piglets were monitored for a period of two months. Of the 7 recipient piglets, one mortality occurred secondary to anesthesia complications while undergoing a routine echocardiogram on post-operative day 19. All piglets had appropriate weight gain and laboratory findings throughout the post-operative period indicating a general state of good health and rehabilitation after undergoing PHT. We conclude that PHT has good short-term survival in the swine model. PHT appears to be safe for clinical application.

## Introduction

Congenital heart disease (CHD) consists of a wide spectrum of cardiac defects that affect approximately 1 in 1000 children born each year^1^. Surgical repair is the first choice for malformations of the heart valves but many infants with CHD ultimately require valve replacement^2,3^. Current options for valve replacement include mechanical or decellularized bioprosthetic valvular implants^4^. However, as neither of these consist of living tissue that can grow, children with valvular implants are often subjected to multiple re-operations until they reach adulthood^4,5^. Partial heart transplantation is an innovative new procedure that involves transplanting only the part of the heart containing the valves^6^. While this technique comes with the standard risks of cardiac surgery, the avoidance of transplanting the entire heart, as would occur in orthotopic heart transplant (OHT), eliminates the risk for major complications like ventricular graft failure, while delivering growing heart valves.

The concept of PHT was first evaluated in heterotopic rodent models^7^. However, it is critical to investigate the safety of orthotopic PHT prior to wide-spread clinical application. Large animal models, including swine, provide important insight into medical advances due to their anatomic and physiologic similarities to humans^8^. As such, results from these models allow for safe translation of findings from the laboratory to patient care. To adequately assess PHT, we have pioneered PHT in piglets. Here, we present the safety and short-term outcomes of PHT in our piglet model.

## Materials and methods

This study was approved by the Committee of Animal Research following the National Institutes of Health Guide for Care and Use of Laboratory Animals (Duke IACUC Protocol ID A052-22-03). All experiments were performed in accordance with the guidelines and regulations laid out by the Duke IACUC Committee. All authors complied with the ARRIVE guidelines. Key portions of the operations were performed by experienced cardiac surgeons.

### Animals

Neonatal Yorkshire pigs between 3 and 7 weeks of age were obtained from different vendors including Wesley Looper Farms (Granite Falls, NC), Valley Brook Research Incorporated (Madison, GA), and National Swine Resource and Research Center (Columbia, MO). Both female and male animals were used for the experiments presented in this study. Recipients and donors were screened for both blood and swine leukocyte antigen (SLA) types. SLA testing was performed as previously described^9^. Pairs were matched based on blood type to simulate clinical PHT.

### Donor operation

For the donor procedure, the animal was premedicated with intramuscular ketamine and acepromazine. General anesthesia was then induced with inhaled isoflurane and intravenous lines were placed. Transthoracic echocardiography was used to assess the donor pulmonary valve for suitability prior to transplantation. If the valve was deemed acceptable, the animal was prepared for surgery. A median sternotomy was performed to gain access to the mediastinum and the heart was exposed. The main and branch pulmonary arteries were carefully dissected out in preparation for procurement. The donor was then systemically heparinized, cannulated via the right atrium and connected to the cardiopulmonary bypass (CPB) pump sucker. The donor piglet was exsanguinated through the pump sucker to prime the recipient CPB circuit. The heart was then recovered, allowing for explantation. Careful dissection of the explanted donor heart was performed, and the pulmonary valve and root were isolated and stored in ice-cold preservation solution while awaiting recipient PHT.

### Recipient operation

The recipients were pre-medicated with amiodarone, antibiotics and induction immunotherapy. The recipient piglet underwent the same preoperative preparation and anesthesia induction as the donor. Analgesia was provided with fentanyl. A midline incision was performed from the thyroid cartilage to the xyphoid process. The carotid artery and internal jugular vein were exposed and cannulated for intraoperative hemodynamic monitoring. A median sternotomy was then performed and a pericardial well created for optimal exposure. The recipient was then systemically heparinized and CPB initiated via aortic and right atrial cannulas. The main pulmonary artery was circumferentially dissected, and the native pulmonary valve resected. The donor graft was then positioned anatomically for partial heart transplantation. The distal anastomosis was created, and the graft was filled retrograde to test the valve for competency. Once this was ensured, the proximal anastomosis was completed, and the recipient was weaned off bypass. Hemostasis was obtained, the sternotomy was closed, and multi-modality analgesia was provided followed by extubated.

### Postoperative management

**Figure 1:**
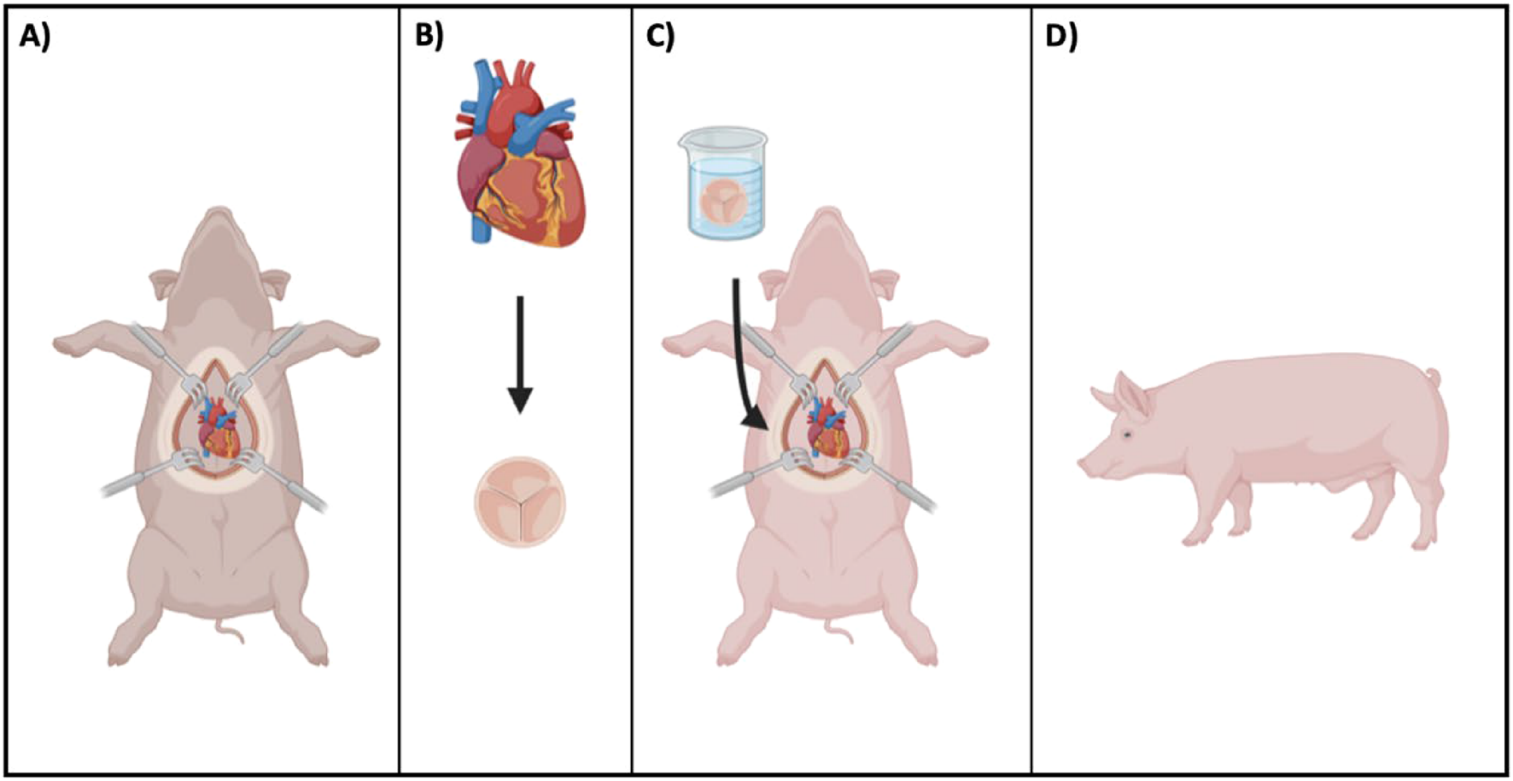
Graphic of experimental design: (**A**) Harvesting of the donor heart (**B**) dissection of the pulmonary valve (**C**) partial heart transplant into recipient (**D**) growing piglet after transplant.

Immediately after the surgical procedure, piglets were taken to their recovery cages where they received pain management. Postoperative monitoring involved routine postoperative checks every 2 h for the first 24 h, which were then extended to every 6 h on post-operative day 2 and every 12 h for the remainder of the experiment. The study animals were immunosuppressed using mycophenolate mofetil and tacrolimus, along with methylprednisolone. Serum immunosuppressive agent levels were measured at least twice weekly. Postoperative labs included weekly hematocrit and creatinine. Antibiotics and peptic ulcer prophylaxis were provided with ceftiofur and omeprazole, respectively. A routine echocardiogram was performed postoperatively. Piglets were followed for approximately 2 months until they had doubled in weight after surgery to ensure adequate assessment time (Fig. 1).

### Statistical analysis

Categorical variables were presented as percentage (number) and continuous variables were presented as mean (range). Survival analysis was performed using Kaplan–Meier methodology. Statistical analysis was performed using GraphPad Prism (version 10.1.1 for Mac OS, GraphPad Software).

## Results

### Baseline characteristics

Our cohort consisted of 7 donor piglets and 7 recipient piglets. Piglets were taken to the operating room when they were approximately 10 kg, which is comparable in size to human children undergoing pulmonary valve replacement. Recipient animals comprised of four females and three males. The mean age and weight at time of PHT was 44 days (range 34–53 days) and 11.3 kg (range 9.1–14.0 kg), respectively (Table 1).

**Table 1:** Baseline characteristics of recipient piglets undergoing partial heart transplantation.

Piglet number	Sex	Age (days)	Weight (kg)
1	F	35	10.6
2	F	51	13.2
3	M	49	9.1
4	M	53	10.6
5	F	40	10.1
6	F	47	11.2
7	M	34	14.0

### Morbidity

**Figure 2:**
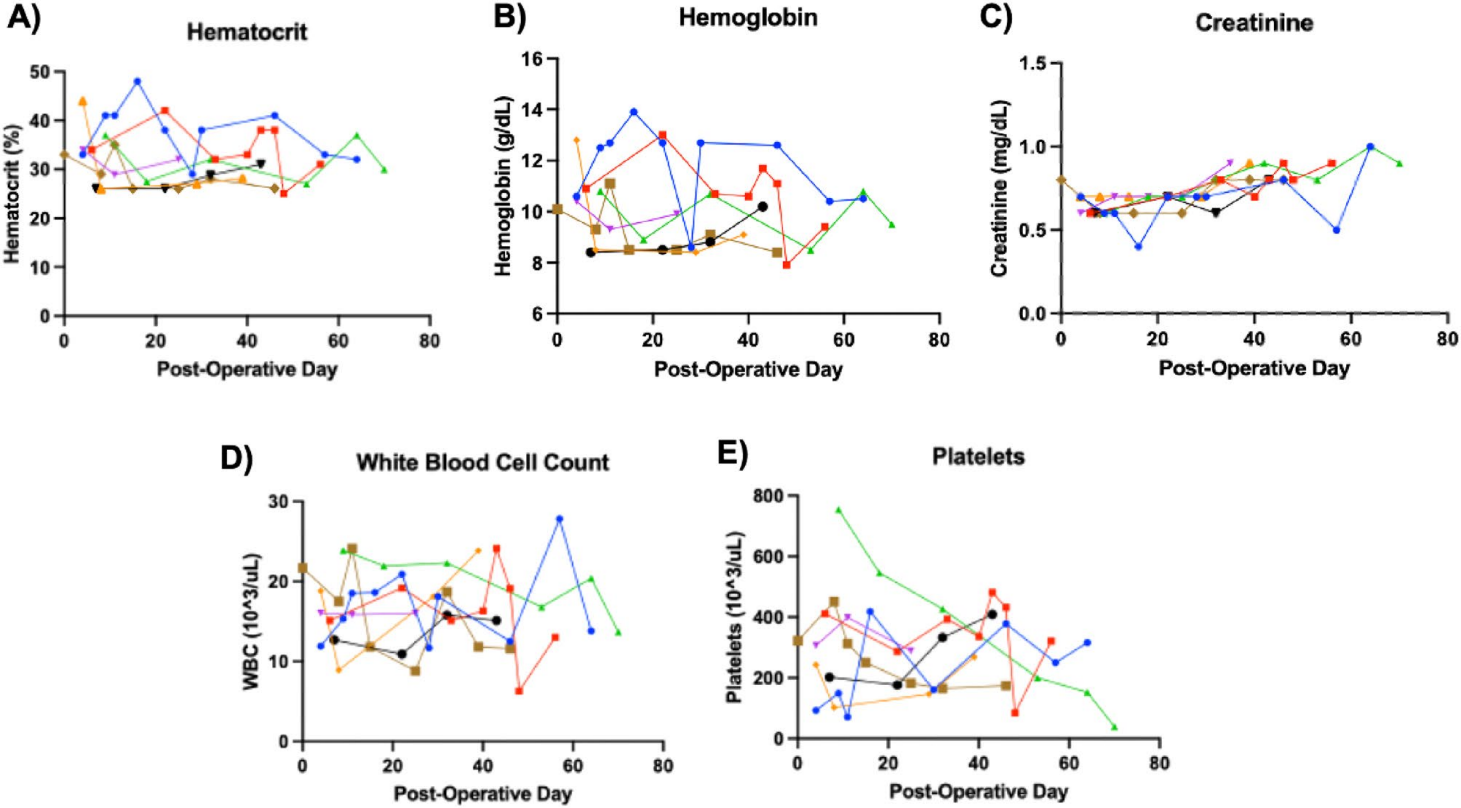
Trend in laboratory values after partial heart transplant: (**A**) hematocrit (**B**) hemoglobin (C) creatinine (**D**) white blood cell count (**E**) platelets.

**Figure 3:**
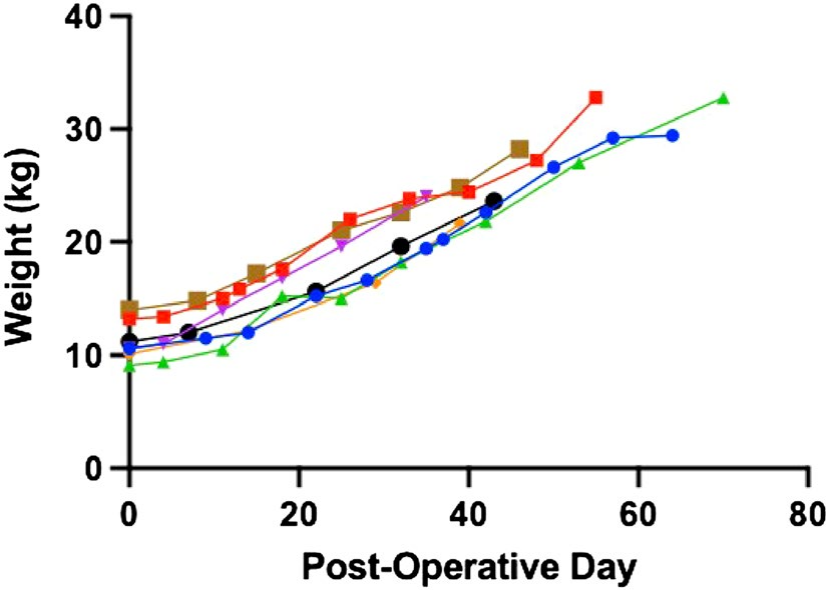
Trend in piglet weight gain across duration of study.

On average, animals voided and had a bowel movement within 2–6 h of surgery. Normal activity levels and food intake were achieved by post-operative day two. Given that surgical blood loss can be a significant stressor and affect recovery after surgery, hematocrit levels were monitored post-operatively (Fig. 2A). After undergoing PHT, routine labs demonstrated appropriate recovery and maintenance of hematocrit after surgery. Similarly, hemoglobin levels recovered and remained stable throughout the study period (Fig. 2B). Creatinine, a surrogate parameter for renal failure^10^, was trended throughout the study (Fig. 2C). Lastly, white blood cell and platelet counts were monitored closely in the early post-operative period (Fig. 2D,E). Study animals doubled their weight in approximately two months, which is in line with previously described weight trajectories of healthy piglets (Fig. 3)^11^.

### Mortality

**Figure 4:**
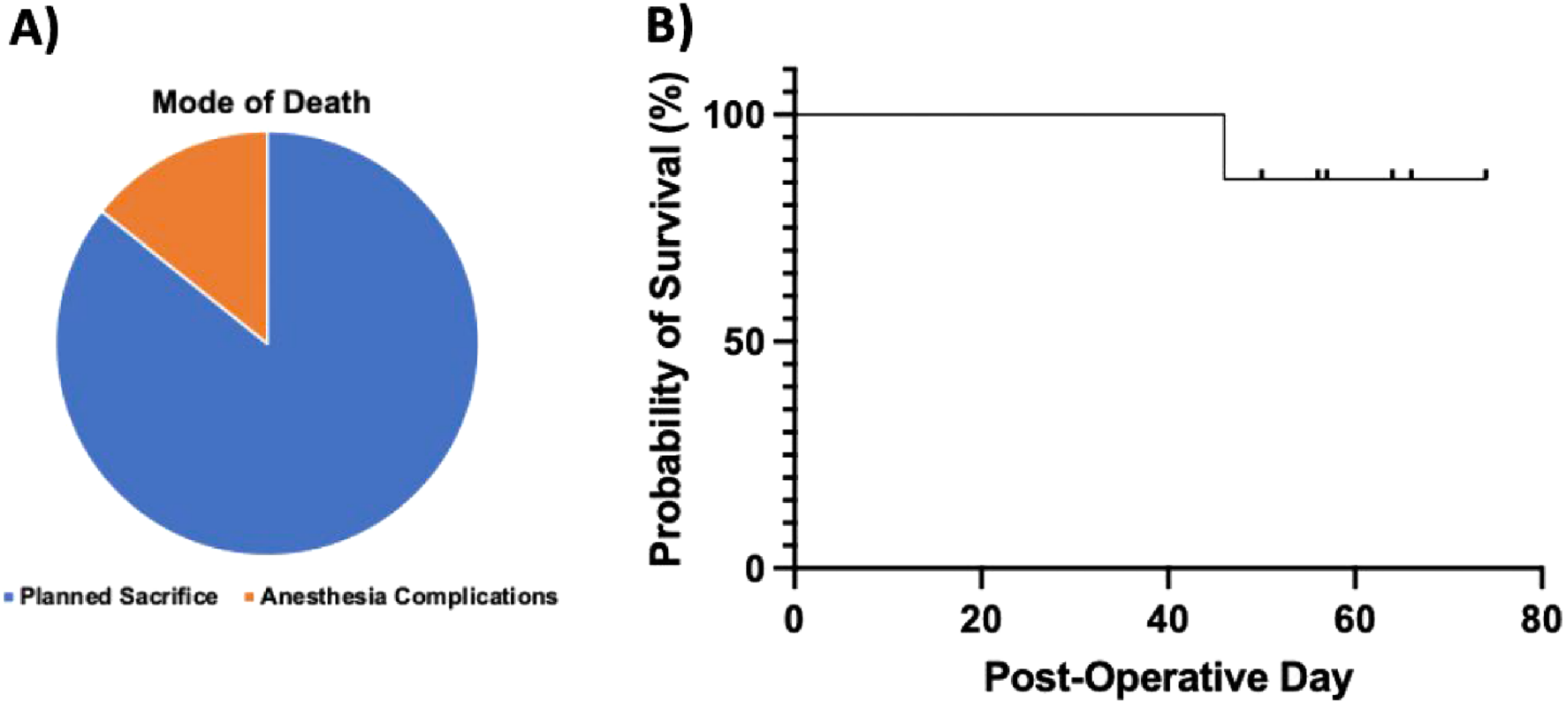
(**A**) Pie chart of mode of death (**B**) Kaplan–Meier survival analysis of piglets undergoing partial heart transplantation.

The overall mortality for recipient animals undergoing PHT was 14.3% (1/7). Of note, no intraoperative deaths were observed. The single mortality occurred on post-operative day 19 and resulted from complications related to anesthesia during a routine echocardiogram (Fig. 4A; Supplemental Table 1). Kaplan–Meier survival analysis of piglets undergoing PHT is presented in Fig. 4B.

## Comment

In this study, we present survival and short-term outcomes of piglets undergoing PHT of the pulmonary valve. In total, 7 piglets received PHT with only one mortality due to anesthesia complications during a routine echocardiogram.  The piglets exhibited appropriate weight gain and laboratory findings throughout the post-operative period, indicating appropriate recovery after undergoing PHT. To ensure sufficient follow-up time after surgery, study animals were monitored until they had doubled in weight, which was for a period of approximately two months. During this time, piglets remained healthy and did not have any operative complications. Taken together, our results indicated that PHT is a safe surgical technique.

### Importance of animal model

While there are other large animal models available to examine the safety and feasibility of PHT, we have chosen the swine model for the experiments presented in this study. The swine model is one of the more popular large animal models for experimental congenital cardiac surgery^12,13^. The anatomic structure of the swine heart and its physiology are akin to that of humans, making them an ideal translational model^14^. With these similarities, piglets are well suited for the use of standard pediatric CPB, enabling researchers to perform the same operative procedures that would be done in human neonates. In addition, the piglet’s immune system mimics that of humans, allowing for the use of the same immunosuppressive agents across species^15^. Lastly, piglets grow quickly and are able to transition from infancy to adulthood in a matter of months, making them a particularly useful model in congenital heart surgery^8^. While sheep are another model commonly used in translational cardiac research, their anatomic variations from pigs pose technical challenges for cardiac surgery: Firstly, the sternum in sheep is narrow, making sternotomies more difficult compared to their swine counterparts^16^. Additionally, sheep have a short ascending aorta^16^, making cannulation onto cardiopulmonary bypass complex, often resulting in exposure of the descending aorta for arterial access. Lastly, a fast-growing model is particularly important to study the transition from infancy to adulthood. Sheep tend to have slower rates of growth, requiring studies to have longer follow-up periods in CHD research^17^.

### Valve transplantation choice

While PHT can occur using any combination of aortic and pulmonic valves in either position, the pulmonary valve transplanted into its anatomic position was particularly well-suited for the swine model. The more anterior positioning of the pulmonary valve allows for less extensive dissection and minimal cardiac manipulation compared to the aortic valve^18^. Importantly, swine hearts are particularly arrhythmogenic when extensively manipulated^14^. Additionally, by transplanting solely the pulmonary valve, cardioplegic arrest of the heart can be avoided, thus allowing for a quicker recovery. Finally, transplantation of the pulmonary valve is less technically demanding given that reimplantation of coronary buttons is avoided^19^, therefore minimizing the risk for ischemia. For these reasons, we have decided to focus on the pulmonary valve to demonstrate the safety and feasibility of PHT.

### Mortality assessment

Out of the 7 recipient piglets that underwent partial heart transplantation, the only mortality occurred during sedation for a routine echocardiogram. Echocardiograms were obtained via a transthoracic approach, which necessitated sedation of the piglets. While under sedation for the echocardiogram, the piglet was no longer able to protect its airway, aspirated, and died shortly afterwards. This incident occurred early in the study protocol and as such, the amount of sedation provided to piglets during echocardiograms was still being optimized.

### Risks and expected complications

While PHT is overall safe, there are some risks that can be expected. Although arrhythmias can lead to intraoperative complications, we have been able to mitigate the risk for severe arrhythmias by premedicating with amiodarone. During PHT, the mediastinum must be entered. While care is taken to avoid entering the pleural space, cases in which the pleural space was violated, a chest drain must be placed to prevent pneumothoraxes. Prior to waking the piglets from general anesthesia, care must be taken to ensure the sternum and soft-tissue incision are closed properly. Given that piglets are quadrupeds, the sternum becomes weight-bearing once the piglets begin walking after surgery. If the chest is not closed properly, sternal dehiscence may occur. Similarly, the soft tissue above the sternum must be closed and topped with a layer of skin glue. Animal housing does not allow for a pristine post-operative environment and the incision often comes in contact with fecal matter when the animal lays down. To prevent wound infection, skin glue is used to provide a physical barrier. As piglets are waking from anesthesia, they must be monitored closely to prevent aspiration. In cases of suspected aspiration, animals must be observed for signs and symptoms of pneumonia, which can be treated with antibiotics and antipyretics. Lastly, it is important to diurese the piglets post-operatively since piglets do not tolerate pulmonary edema^20^.

### Clinical translation

Our study demonstrates the overall safety and feasibility of PHT in a large animal model. This pre-clinical work led to our center performing the world’s first human PHT^21^. As this procedure gains traction in the field of pediatric cardiac surgery, just allocation of donor grafts will become an issue because pediatric donor hearts are a scarce resource. Children in need of a conventional heart transplant have no alternative treatment options as ventricular assist devices suitable for destination therapy are only available at adult size. In contrast, children in need of a PHT who decompensate waiting for a PHT can reasonably be treated with a non-growing homograft. Therefore, children awaiting partial heart transplantation should not compete with patients urgently awaiting a conventional hear transplant.

Importantly, evidence suggests that every year, there are pediatric hearts which are not utilized for conventional heart transplant as they are deemed low likelihood for transplant success^22,23^. We estimate that approximately one third of pediatric donor hearts are unsuitable for full organ transplantation and could be used for PHT^24^. Thus, PHT may represent an avenue for improved organ stewardship, by allowing for use of donor hearts that may otherwise be discarded.

## Limitations

Our study examined PHT of the pulmonary valve in a piglet model. The pulmonary valve was chosen because it is a technically easier operation. However, the design chosen for this study does not allow for examination of hemodynamic perturbations that can be expected with PHT of the aortic valve. Finally, although the immune systems of pigs are similar to that of humans, there are species-specific differences. As such, the efficacy of the immunosuppressive medication regimen used in human neonates and potential immunomodulatory effects remain not fully understood.

## Conclusions

Through the use of a swine large animal model, partial heart transplantation of the pulmonary valve has been shown be a safe and effective alternative to mechanical or biologic valve replacement. Animals undergoing pulmonary valve PHT have demonstrated good short-term survival and outcomes. These results not only demonstrate the feasibility of PHT but also indicate that it can be safely performed in humans.

### Supplementary Information

Supplementary Information.

## Data Availability

All data generated or analyzed during this study are included in this published article (and its Supplementary Information files).
