# Supplementary material for: Survival after partial heart transplantation in a piglet model
Source: Sci Rep. 2024 May 29;14:12318. doi: 10.1038/s41598-024-63072-1 (PMC11136985; doi:10.1038/s41598-024-63072-1)
Supplement: Supplementary file 1 — Supplementary Information. [file 41598_2024_63072_MOESM1_ESM.docx]

**SUPPLEMENTAL TABLES**

**Supplemental Table 1:** Modes and Time of Death in Piglets Undergoing Partial Heart Transplantation

| **Piglet Number** | **Mode of Death** | **Post-Op Survival (days)** | **Premature Death** |
| --- | --- | --- | --- |
| 1 | Planned Sacrifice | 66 | No |
| 2 | Planned Sacrifice | 56 | No |
| 3 | Planned Sacrifice | 74 | No |
| 4 | Anesthesia Complications | 46 | Yes |
| 5 | Planned Sacrifice | 57 | No |
| 6 | Planned Sacrifice | 50 | No |
| 7 | Planned Sacrifice | 64 | No |
